# Nuclear receptor modulation by kinesin

**DOI:** 10.18632/aging.101410

**Published:** 2018-03-30

**Authors:** A.M.P.B. Seneviratne, Geri Kreitzer

**Affiliations:** 1 Department of Molecular, Cellular and Biomedical Sciences, The City University of New York School of Medicine, New York, NY1003, USA

**Keywords:** estrogen related receptor alpha, kinesin, nuclear receptor box, transcription

Estrogen related receptor alpha (ERR1) is an orphan nuclear receptor (NR) that plays a key role in adaptive energy and lipid metabolism, mitochondrial biogenesis, osteogenesis, and in regulation of blood pressure (Huss, JM, et al. 2015). ERR1 interacts with co-factors in a ligand-independent manner and can modulate transcription constitutively, although signal-mediated post-translational modifications and competition with other NRs affect DNA and cofactor binding, providing control points for regulation of ERR1 transcriptional activity. Dysregulated ERR1 is associated with numerous pathophysiological conditions, and interest in targeting ERR1 therapeutically is high. However, as a consequence of its role in controlling multiple critical cellular pathways, there is a need to identify or design agents that impact ERR1 transcriptional outputs selectively. A recent study by Seneviratne et al. reveals that kinesin family motor proteins may serve such a purpose by modulating coactivator binding [1].

Kinesins are evolutionarily conserved microtubule (MT) stimulated ATPases that perform essential functions in cells. The canonical roles of kinesin motors include transport of varied cellular cargoes, regulation of MT dynamics, and chromosome capture and segregation during mitosis. Although less widely appreciated, a growing body of work highlights a non-canonical role for kinesins as regulators of transcription. This unconventional activity was first described for the Drosophila kinesin-related protein costal2, which interacts in a complex with the serine/threonine kinase fused (Fu) and the transcription factor cubitus interruptus (Ci) to modulate transcription in response to Hedgehog (Hh) signaling [2]. Kinesin-like protein KIF7, the vertebrate homolog of costal2, interacts with Suppressor of Fused (SuFu) and Gli, regulating Gli processing and transcriptional function in an Hh-dependent manner [3,4]. A second kinesin, KIF17b, regulates CREM activity in male germ cells by controlling the nuclear-cytoplasmic localization of ACT, an activator of CREM mediated transcription [5]. In both these cases, the kinesin affects transcription indirectly by sequestering transcription factors on MTs in the cytoplasm or cilium to restrict their nuclear function, albeit by distinct mechanisms.

Evidence of a more direct effect of kinesin on transcription was recently demonstrated by Seneviratne et al for KIF17 and ERR1 in breast cancer cells [1]. KIF17 contains a nuclear localization signal, and both endogenous and overexpressed protein localizes to the cytoplasm and the nucleus. An LXXLL NR-box motif, conserved in NR coactivators, is located in the C-terminal tail domain of KIF17 and mediates the interaction of KIF17 with C-terminal half of ERR1, containing binding sites for cofactors and ligands. Expression of a peptide containing the NR box is sufficient to inhibit transcription of a subset of ERR1 target genes (including ERR1 itself), and cell invasion through Matrigel^TM^. Depletion of KIF17 or expression of a KIF17 fragment lacking the NR box (yet retains ERR1 binding) results in increased ERR1 activity in reporter assays. Although the effects of these latter treatments on specific ERR1 targets is not yet known, the nuclear localization of expressed KIF17 peptides suggests they modulate transcription by competing with other ERR1 cofactors for ERR1 binding in the nucleus. Specific amino acids flanking the NR-box may be key in achieving selective cofactor binding and transcriptional response [6].

The finding that expression of KIF17 peptides is sufficient to modify ERR1 activity selectively, regardless of endogenous KIF17, opens a potential advantageous avenue to modulate ERR1 function effectively across a range of disease states. As an example one can consider the impact of ERR1 on metabolism and bone remodeling that accompanies aging. In mouse models, age-associated reduction of ERR1 levels and ERR1-dependent expression of peroxisome proliferator-activated receptor gamma coactivator 1A (PGC1A) and glutaminase results in glucose intolerance and inhibition of osteoblastogenesis [7,8]. PGC1A acts with ERR1 to broadly regulate transcription of mitochondrial genes of metabolism. Glutaminase is required for mitochondrial glutamine-dependent anaplerosis, a process essential for osteoblastic induction of mesenchymal stem cells. By contrast, in models of osteoporosis, MYC induction of ERR1, working with the cofactor nuclear factor of activated T cells c1, drives osteoclastogenesis and bone loss (Bae S, et al. 2017). Selective modulation of ERR1-cofactor interactions, and thus a subset of transcriptional targets, may represent a viable strategy to combat degenerative bone loss and metabolic attenuation associated with aging.

The findings described in Seneviratne et al. do not go as far as identifying which ERR1 cofactors interactions are disrupted by the KIF17 NR-box peptide, but they open a Pandora’s Box of therapeutic possibilities revolving around NR-box motifs. Important next steps include determining which cofactors bind ERR1 in the absence and presence of NR-box peptides, how sequences flanking the NR-box contribute to cofactor-ERR1 binding, and how this affects cellular programming. Of note, LXXLL NR-box motifs are found in additional kinesin family motors, hinting at a heretofore unappreciated mechanism by which to tune nuclear receptor activities in specific cellular contexts.
